# A discussion on the application of fluorescence micro-optical sectioning tomography in the research of cognitive dysfunction in diabetes

**DOI:** 10.1515/med-2025-1254

**Published:** 2025-09-12

**Authors:** Qisheng Liu, Shaobing Dai, Bing Yan, Yutong Gan, Hao Jiang, Yan Chen, Qixuan Li, Lingjie Li, Kaiyuan Zou, Yurong Liu

**Affiliations:** School of Biomedical Engineering and Imaging, Xianning Medical College, Hubei University of Science and Technology, Xianning, Hubei, 437100, China; Department of Gastroenterology, Xianning Central Hospital, The First Affiliated Hospital of Hubei University of Science and Technology, Xianning, Hubei, 437000, China; College of Innovation and Entrepreneurship, Xianning Medical College, Hubei University of Science and Technology, Xianning, Hubei, 437100, China

**Keywords:** diabetic cognitive dysfunction, fluorescence micro-optical sectioning tomography, neural circuits, vascular imaging

## Abstract

**Objectives:**

This review explores the application value of fluorescence micro-optical sectioning tomography (fMOST) in diabetes-related cognitive dysfunction research, emphasizing its unique capacity to resolve microstructural alterations in neural circuits and vascular networks, thereby offering novel insights into the pathogenesis of type 2 diabetic cognitive impairment.

**Methods:**

Existing literature was analyzed to evaluate fMOST’s principles and capabilities, including its achievement of whole-brain three-dimensional imaging at sub-micron resolution, simultaneous acquisition of neuronal morphology (soma, dendritic spines, axonal terminals) and vascular networks, and integration with fluorescent labeling to trace prefrontal cortical pyramidal neuron projections under pathological conditions.

**Results:**

fMOST technology revealed the critical role of neurovascular coupling dysfunction in diabetic cognitive impairment, demonstrating that interactive damage between neurons and vasculature collectively drives disease progression. In type 2 diabetic models, it identified abnormal synaptic structures in prefrontal/hippocampal pyramidal neurons, vascular network remodeling, and disrupted brain connectivity. Compared to conventional imaging (magnetic resonance imaging/positron emission tomography), fMOST enables concurrent quantitative analysis of synaptic-level neural circuits and microangiopathy, overcoming the resolution limitations of macroscopic imaging.

**Conclusion:**

fMOST serves as an indispensable high-precision, multi-scale imaging tool for investigating diabetic cognitive impairment. Future priorities include elucidating dynamic neurovascular unit interactions in diabetic encephalopathy, developing neural circuit-targeted interventions, and advancing interdisciplinary integration to accelerate clinical translation.

## 1 Introduction

Diabetes is a widespread chronic condition affecting metabolism, notable for chronic high blood sugar levels and disrupted processing of carbs, fats, and proteins, due to a deficiency in insulin production, a decrease in insulin effectiveness, or both [1]. However, with the enhancement of average life spans and an upgrade in the quality of life, the occurrence of diabetes is experiencing a yearly escalation. The International Diabetes Federation reports that the worldwide prevalence of diabetes stood at 10.5% in the year 2021, with forecasts indicating an increase to 11.3% in 2030 and 12.2% by 2045. In 2021, diabetes accounted for approximately $966 billion in global health expenditure, constituting 9% of total global health expenditure, and led to the deaths of an estimated 6.7 million people aged 20–79 [2]. Currently, diabetes ranks as the third most serious chronic lifelong disease threatening human health after cancer and cardiovascular and cerebrovascular diseases, posing a significant global public health challenge [3]. In clinical practice, the most commonly utilized and universally recognized classification of diabetes, based on its causes, is the fourfold division established by the World Health Organization in 1999: Type 1 diabetes, type 2 diabetes, unique forms of diabetes, and gestational diabetes. Of these, type 2 diabetes, also referred to as non-insulin-dependent diabetes mellitus, accounts for more than 90 ± 5% of all diabetes cases today [4]. It is marked by resistance to insulin in the body’s peripheral tissues, which induces glucose intolerance and elevated blood sugar levels. With the advancement of the disease, diabetes can precipitate long-term complications that impact various bodily systems, potentially leading to chronic impairment or the outright failure of tissues and organs, including the eyes, kidneys, heart, vascular system, and nervous system. Current research on diabetes-related chronic complications primarily focuses on diabetic cardiomyopathy, diabetic nephropathy, diabetic retinopathy, and other areas. However, the brain, as the advanced neural center, presents unique challenges in functional research, with few studies addressing central nervous system complications of diabetes [5]. Cognitive dysfunction associated with type 2 diabetes encompasses deficits in brain function and cognitive abilities in those affected [6]. Individuals with this condition typically show difficulties in learning and memory, a reduction in the efficiency of executive functions, a decrease in the rate of information processing, as well as neuropathological signs including neurodegeneration and associated anomalies in neurochemistry and neural architecture. The degree of impairment ranges from mild to severe, broadly categorized into three stages: diabetes-related cognitive decline, mild cognitive impairment, and dementia [7]. Studies have confirmed that the incidence of dementia among type 2 diabetes patients aged 60–64 is 0.83%, rising to 10% among those over 85 years old. Due to advancements in medical technology, the lifespan of patients with diabetes-related cognitive impairment has extended, leading to an increasing elderly population with the condition [8]. Diminished self-management capabilities significantly impact patients’ quality of life, while increased dependency on care imposes a heavy burden on society and families [9]. In conclusion, elucidating the pathogenesis of type 2 diabetes-related cognitive dysfunction and developing effective prevention and treatment strategies are urgent priorities [10].

Although there have been numerous studies on the pathological mechanisms underlying diabetic cognitive dysfunction, its complexity poses challenges to a comprehensive understanding, particularly in the in-depth analysis of the microstructure and function of brain tissue [11]. In recent years, multiple studies have reported that disruption of neurovascular coupling may be a potential mechanism underlying cognitive impairment in type 2 diabetes [12]. In 2010, researchers proposed considering neurons and blood vessels as a functional complex, termed the neurovascular unit, which plays a crucial role in maintaining normal brain function by providing sufficient blood to the corresponding neurons [13]. However, in type 2 diabetes, insulin resistance or insulin deficiency maintains a state of hyperglycemia, leading to non-enzymatic glycation of structural and functional proteins, nucleic acids, and other proteins in the body. This eventually forms irreversible advanced glycation end product, whose neurotoxic effects may disrupt neurovascular coupling, causing disorders in cerebral microcirculation, imbalance in blood supply, and chronic hypoperfusion of brain tissue. This leads to ischemia, hypoxia, and neuronal death, which is often considered the primary cause of cognitive impairment and even Alzheimer’s disease [14]. Thus, it is evident that dysfunction between neurons and blood vessels can influence each other and may both contribute to the occurrence of cognitive impairment in type 2 diabetes [15]. Diabetes is often accompanied by cerebral vascular injury, particularly affecting microvasculature and small vessels. This primarily involves alterations in the properties and functions of microvasculature and small vessels, reductions in cerebral vasodilatory activity, and changes in cerebral blood flow. These changes often precede the onset of diabetes. Experimental studies in animals have demonstrated the presence of neovascularization and remodeling of the existing vascular system in various diabetic models, including GK rats, db/db mice, and HFD/STZ models. These newly formed and remodeled vessels are immature, hypoperfused, and pathological. All of these changes can potentially lead to disorders in cerebral microcirculation, resulting in inadequate perfusion of brain tissue in diabetic patients [16]. Undoubtedly, adequate blood flow is crucial for maintaining normal brain structure and function. While some articles have reported a positive correlation between diabetic cognitive impairment and cerebral microvasculopathy, researchers have found that patients still exhibit lesions in brain tissues related to cognitive function after excluding the factor of cerebrovascular disease. Functional magnetic resonance imaging (fMRI) has revealed white matter lesions in type 2 diabetes patients, manifested as increased signal intensity, disrupted integrity, and reduced functional connectivity. Furthermore, these white matter abnormalities in the left and right hemispheres are asymmetric, with abnormalities in the left hemisphere being more common [17]. In recent years, an increasing number of studies have shown that gray matter lesions occur in brain regions such as the prefrontal cortex, hippocampus, amygdala, insula, cingulate gyrus, caudate nucleus, and thalamus in type 2 diabetes patients. These lesions may serve as the anatomical basis for anxiety, depression, and cognitive impairment in type 2 diabetes patients [18]. The question of whether white matter or gray matter abnormalities are the neuropathological substrates for cognitive deficits in type 2 diabetes continues to be a topic of debate. Research suggests that compromised integrity of white matter in the right fronto-occipital fasciculus correlates with impairments in episodic memory and attention, and disruptions to white matter in the corpus callosum, the left anterior limb of the internal capsule, and the external capsule are tied to reduced executive functioning. Nevertheless, findings from a prospective cohort study suggest that white matter abnormalities may not be the primary pathological alterations leading to cognitive decline in type 2 diabetes. Regarding the involvement of gray matter in cognitive deficits associated with type 2 diabetes, there is ongoing discussion about its contribution to the impairment of learning and memory. The existing evidence points to broad and non-specific alterations in the vasculature and brain structure in type 2 diabetes, yet the precise connection between these changes and cognitive dysfunction in this condition remains indistinct and calls for additional research [19].

Currently, the primary clinical methods used to investigate the relationship between changes in brain neural circuits and vascular networks and cognitive deficits include positron emission tomography, structural and functional magnetic resonance imaging, and diffusion tensor imaging (DTI). In addition, their primary advantages lie in their abilities to achieve non-invasive *in vivo* imaging with short acquisition times for large biological tissues such as the brain [20]. However, their drawbacks include relatively low resolution, which prevents them from reflecting brain structural and functional activities at the neuronal resolution level, essentially “blurring” the image.

In recent years, thanks to advancements and applications in fluorescent labeling technology, optical microscopy imaging has emerged as a powerful tool for studying the structure of brain neural circuits and vascular networks [21]. Optical microscopy imaging techniques typically allow for imaging of biological samples on the centimeter scale with high resolution. Notably, the establishment of micro-optical sectioning tomography (MOST) has enabled the systematic construction and identification of detailed cerebrovascular maps encompassing arteries, veins, arterioles, and venules throughout the entire brain. In conclusion, building upon MOST, the fluorescence micro-optical sectioning tomography (fMOST) technique has been developed, capable of capturing neuronal fine structures such as neuronal cell bodies, dendritic spines, and synaptic boutons at sub-micrometer resolution [22].

**Figure 1:**
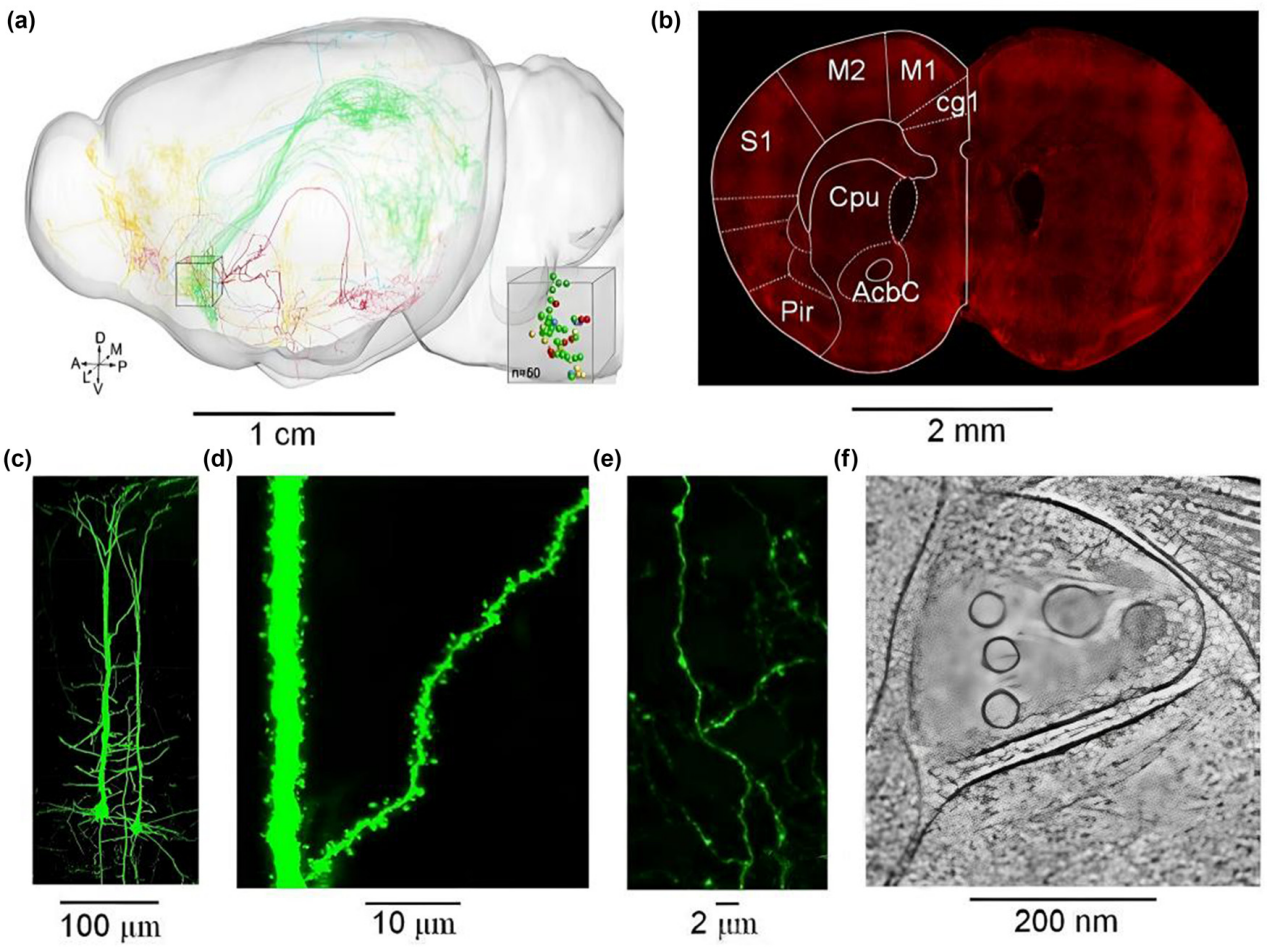
Comparison of neural circuits at different research scales (a–f).

Neuronal axon fibers can reach a length of centimeters and span multiple brain regions, as shown in Figure 1. The fine scale can reach the connection point of neural circuits – the synaptic junction (bouton), with a diameter of 1–2 μm.

In both clinical populations and animal studies, structural deterioration like neuron loss tends to manifest in the mid to advanced phases of the disease. Conversely, cognitive impairment observed in the early phases is often attributed to dysfunctional synaptic activity within certain brain regions, notably the prefrontal cortex and hippocampus. The prefrontal cortex is acknowledged as a pivotal brain region responsible for sophisticated cognitive processes, such as emotional regulation, working memory, decision-making, motor planning, and executive functions [23]. Neurons in the prefrontal cortex send projections extensively throughout the brain, encompassing nearly all areas, including the cortex, striatum, thalamus, midbrain, and hindbrain. Abnormalities in its structure and function can lead to various cognitive impairment-related diseases. Although studies have reported changes in the prefrontal cortex in diabetic models, most of the focus has been on alterations in macroscopic structures such as gray matter or white matter, with minimal simultaneous investigation of changes in neurons and blood vessels.

In summary, utilizing fMOST imaging technology to acquire whole-brain datasets and analyze the output circuits of pyramidal neurons in the prefrontal cortex as well as vascular networks, and comparing the differences in neural circuits and vascular networks under pathological and physiological conditions, can provide a new perspective for exploring the pathogenesis of cognitive dysfunction in type 2 diabetes and theoretical support for early diagnosis and treatment [24].

## 2 Overview of fMOST

In 2010, the MOST system, developed by Professor Luo Qingming’s team at Huazhong University of Science and Technology, leveraged an imaging system to capture images of the sample surface, while a microtome removed superficial tissues from the plastic-embedded sample, allowing for imaging of the newly exposed surface. This process culminated in the acquisition of complete mouse brain data with an axial resolution of 1 μm [25]. Targeting fluorescently labeled specimens, Professor Luo’s team further developed a series of fMOST systems. This technology not only enables imaging of large-scale whole-brain samples in mice but also boasts advantages of three-dimensional high resolution and inherently registrable data, making it the most promising imaging technique currently available for tracing the complete projection pathways and connectivity of fluorescently labeled neurons throughout the entire mouse brain [26]. fMOST enables rapid high-resolution imaging throughout the entire brain, achieving a spatial resolution of the sub-micrometer level. It can comprehensively image mouse brains exceeding 1 cm^3^ in size. The data acquisition process is fully automated, and the acquired tomographic data undergo automatic registration, satisfying not only the need for imaging at the neural circuit level but also the acquisition of detailed neuronal morphologies, laying a solid foundation for studying neural circuit development. Through this technology, researchers can achieve high-resolution three-dimensional imaging of brain regions associated with cognitive impairment in diabetes, gaining insights into potential changes in diabetic brains, such as neuronal loss and synaptic alterations. This information contributes to unraveling the specific mechanisms underlying cognitive decline and provides a scientific basis for developing more effective intervention strategies [27].

**Figure 2:**
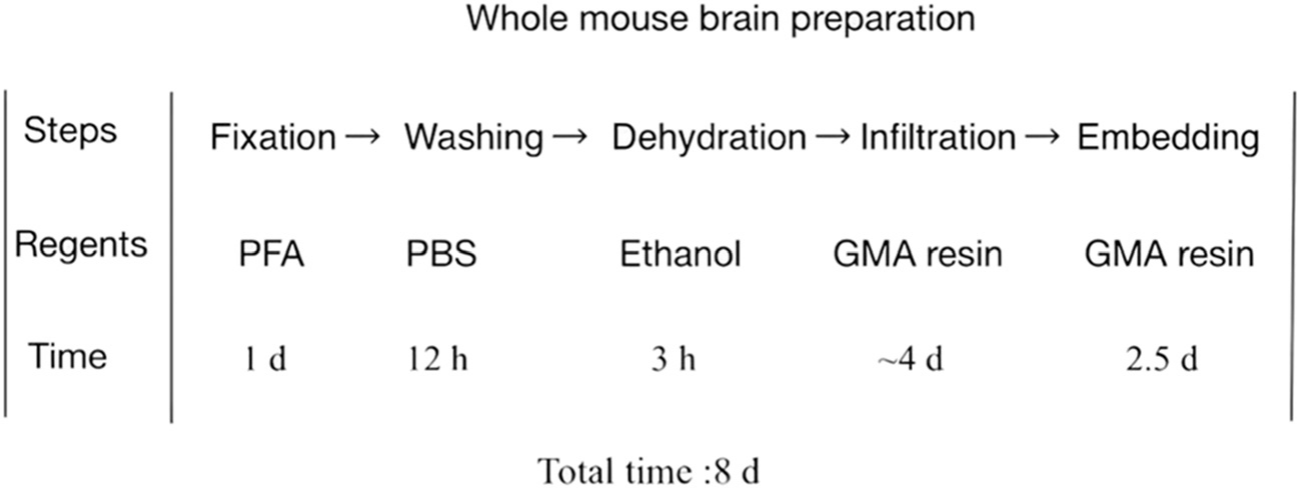
Production process of mouse whole brain samples.

The whole brain sample preparation process of transgenic mice labeled with fluorescent protein is shown in Figure 2, which mainly includes fixing, rinsing, and washing. Dehydration, infiltration, embedding, and polymerization are six steps, and it takes 8 days to complete all operations.

## 3 Application of fMOST in studying neural circuits in mice

### 3.1 Neuron tracing

The fMOST technology, with its axial resolution of 1 μm and the capability of continuous slicing throughout the entire brain, offers a pathway for studying neuronal morphology and neuropathological relationships. This technology integrates high resolution with large-scale acquisition capabilities, enabling high-definition scanning of centimeter-scale brain tissues [28]. Neuron tracing is achieved through the use of fluorescent probes that label neurons. This labeling can be performed at the single-cell level or at the population level of neurons. By reconstructing neural circuits across the entire mouse brain and tracing the distribution and connectivity patterns of neurons, researchers can gain a deeper understanding of the structure and function of neural circuits [29].

### 3.2 Synapse imaging

The fMOST technology not only enables observation of neuronal distribution but also facilitates synapse imaging. By labeling synapse-related proteins or fluorescently tagged postsynaptic structures, researchers can investigate synapse morphology, density, and function. This technology reconstructs the dendritic morphology of long-range projecting neurons and maps the axons of multiple neurons within a single mouse brain, facilitating subsequent analyses of neuronal dendritic morphology [30,31].

### 3.3 Functional connectivity

The fMOST technology, by integrating the strengths of fluorescence microscopy and optical sectioning imaging, enables the observation of functional connections between neurons [32]. Researchers can utilize functional fluorescent probes or optogenetic techniques to observe signal transmission and connection patterns between neurons in real-time. This approach provides dynamic recordings of neural circuit activities, thereby offering profound insights into the functional connectivity within the nervous system [33].

**Figure 3:**
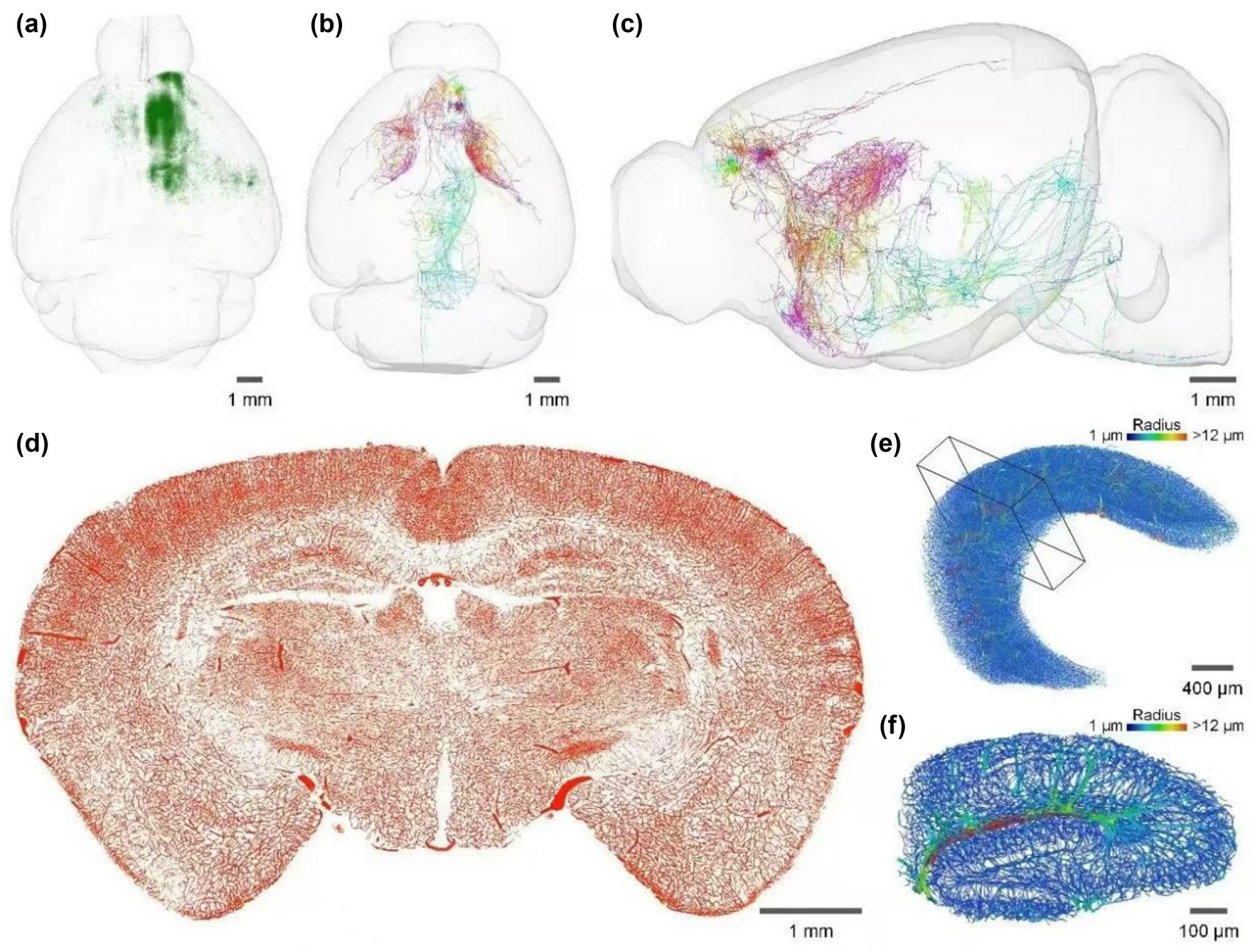
Display of whole brain microscopic optical imaging results (a–f). Reproduced with permission from ref. [34]. Copyright 2023, Neuroscience.

Figure 3 provides the whole brain microscopic optical imaging results. (a) Three-dimensional horizontal rendering of mouse whole brain with single-stage input neurons. (b) and (c) Horizontal and sagittal display of neuronal morphology reconstruction results. (d) and (e) Three-dimensional visualization results of local blood vessels in hippocampus.

## 4 Application prospects of fMOST in diabetic cognitive impairment

The application of fMOST in revealing the neuronal structures and connections in the brains of diabetic patients offers a high-resolution, three-dimensional imaging approach, facilitating a profound understanding of neuronal alterations in diabetic cognitive impairment [35]. By utilizing fluorescent proteins to label neuronal nuclei, axons, dendrites, and other structures, fMOST enables high-resolution imaging of neuronal architectures within brain tissues. In the study of neuronal structures in diabetic brains, fMOST allows for the observation of detailed morphological features such as neuron shape, size, and branching patterns, facilitating comparisons between normal and impaired tissues.

Furthermore, fMOST technology can illuminate the synaptic connections between neurons. Synapses, crucial structures for signal transmission between neurons, exhibit morphological and quantitative changes that may correlate with cognitive decline. Through fluorescent labeling of synaptic proteins, researchers can perform quantitative and localized imaging of synapses, further analyzing morphological alterations and density reductions in diabetic brains. The location of neurons within brain regions is intimately tied to their function [36]. To comprehend the role of neurons within brain networks, it is essential to accurately map their positions. Additionally, acquiring information on vascular network structures is vital for investigating the pathogenesis of diabetic cognitive impairment. Currently, a whole-brain cellular fluorescent restaining method for real-time tissue surface staining enables the simultaneous acquisition of neuronal morphology and co-localized three-dimensional cytoarchitectural background information for neuron localization. In conclusion, by selecting appropriate dyes, it is possible to concurrently capture information on cellular architecture and vascular network structures. Our research group is currently engaged in this endeavor.

**Figure 4:**
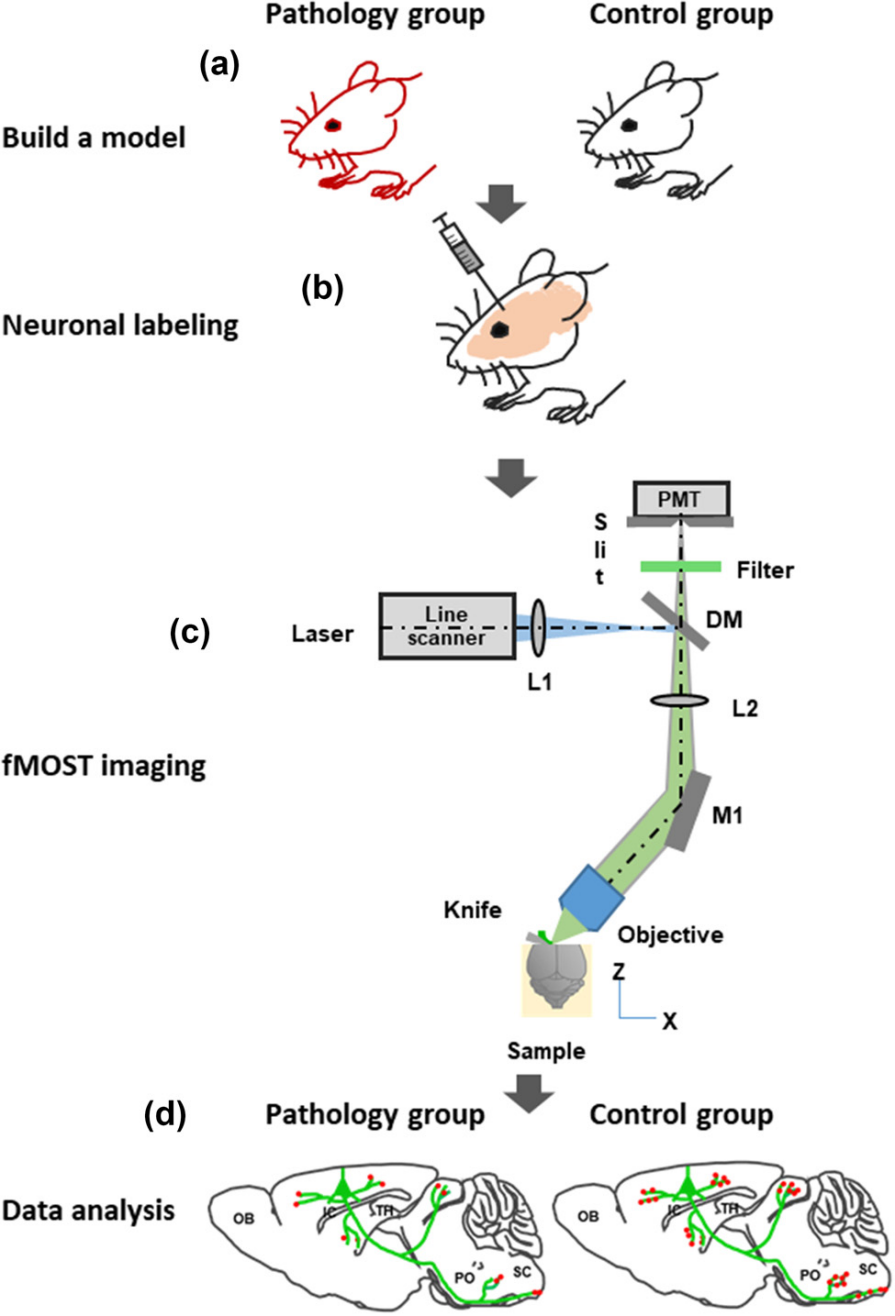
Technical route of the project (a–d).

As shown in Figure 4, diabetic model group and physiological control group were set. (a) The mental state, fur gloss, water intake, and urine volume of the mice were observed and recorded, and the changes of body weight and blood sugar of the mice were detected. (b) The morphology and presynaptic structure of pyramidal neurons in the prefrontal and hippocampal brain regions were marked with whole-brain, sparse, high-brightness. (c) Two-color fMOST imaging and the vascular network structure was obtained simultaneously. (d) At the synaptic level, a quantitative and systematic analysis of the differences in the projection of prefrontal neurons in pathological and physiological model states and changes in vascular networks in the two models was performed for the pathological and control groups.

Neural circuit changes in diabetic cognitive impairment follow a specific pathological sequence. Initially, hyperglycemia and metabolic disorders lead to neuronal apoptosis and injury, which are early manifestations of neural circuit damage. Subsequently, the reduction in neural development further exacerbates the structural abnormalities of neural circuits. During this process, abnormal autophagy function disrupts the homeostasis of neurons, accelerating neuronal damage. In particular, the hippocampus, a key brain region for learning and memory, exhibits pronounced neuronal changes, directly impacting cognitive functions. Eventually, these pathological changes converge to cause disorders in neural signal transmission, resulting in functional impairments such as cognitive decline. fMOST technology can provide high-resolution three-dimensional imaging for each of these stages, offering researchers a powerful tool to explore the pathogenesis of diabetic cognitive impairment. By capturing the dynamic changes of neural circuits from cellular injury to functional impairment, fMOST helps reveal the specific mechanisms of cognitive dysfunction in diabetes, providing a theoretical basis for early diagnosis and treatment.

In summary, fMOST technology not only enhances our understanding of the pathogenesis of diabetic cognitive impairment but also provides new ideas for the development of related drugs. Its application prospects are vast.

## 5 Research prospects

By adopting the fMOST technology, researchers can delve into the roles of neuronal structures and connections, neuroprotective mechanisms, and other factors in diabetic cognitive impairment, providing crucial clues for understanding the disease’s pathogenesis and treatment. Specifically, the application of fMOST in the study of diabetic cognitive impairment encompasses the following aspects:

(1) Unveiling disease mechanisms: This technology aids researchers in gaining a profound understanding of the mechanisms underlying neuronal damage, alterations in synaptic structures and numbers, and other factors in diabetic cognitive impairment. It provides intuitive and detailed observational and analytical data for the onset and progression of the disease.
(2) Discovering potential therapeutic targets: By studying changes in the structures and synapses of specific brain regions or neuron types, researchers can identify new potential therapeutic targets, thereby offering more options for developing treatment strategies for diabetic cognitive impairment.
(3) Enabling quantitative analysis and three-dimensional imaging: The development of this technology enables researchers to conduct precise quantitative analysis and three-dimensional imaging, providing a more comprehensive understanding of disease-related changes at the tissue structure and cellular levels. This offers a deeper perspective for research.

In the future, the field of diabetic cognitive impairment still faces several challenges and development directions for fMOST technology:

(1) Technological improvements: Further enhancing imaging resolution, reducing imaging time, and improving signal detection sensitivity will strengthen the application effectiveness of this technology in disease research.
(2) Data analysis and integration: Strengthening the analytical techniques and integration capabilities of fMOST data, leveraging bioinformatics and artificial intelligence, can aid in better comprehending the complex mechanisms of the disease.

To further investigate diabetic cognitive impairment, it is recommended that future research should reinforce interdisciplinary collaboration, integrating professional knowledge and technological means from different fields to jointly explore the disease’s pathogenesis and novel treatment avenues. Additionally, it is essential to enhance the integration of clinical and basic research, translating laboratory findings into clinical applications to provide patients with more effective diagnostic and treatment options. Comprehensive utilization of modern technological means to continually promote the application of fMOST technology in diabetic cognitive impairment research, which holds promise for a deeper understanding of disease mechanisms, the development of new treatment strategies, and ultimately, bringing benefits to patients.

## 6 Conclusions

This review integrates the association between Type 2 diabetes mellitus brain structural damage (such as atrophy and white matter lesions), neurological dysfunction (such as decreased synaptic plasticity), and imaging techniques (such as DTI), providing a technical pathway for early identification of cognitive impairment through imaging markers. The fMOST technology enables deep analysis of neurological changes: fMOST provides high-resolution, three-dimensional structural information of the nervous system, enabling researchers to gain insights into the changes in neuronal connections and damage in the brains of patients with diabetic cognitive impairment. This technology offers intuitive observation and quantitative analysis for understanding the disease’s development mechanisms. Furthermore, it can assist in discovering new therapeutic targets, providing crucial clues for developing novel treatment strategies, and potentially facilitating innovation and progress in disease treatment. The application of this technology will help advance research in the field of diabetic cognitive impairment, promote interdisciplinary collaboration and technological innovation, and present new opportunities and challenges for cross-disciplinary research in neuroscience, diabetology, and imaging science.
